# Retroperitoneal Metastasis Abutting Small Bowel: A Novel Magnetic Resonance-Guided Radiation Approach

**DOI:** 10.7759/cureus.2412

**Published:** 2018-04-02

**Authors:** Ahmed I Ghanem, Carri Glide-Hurst, M.Salim Siddiqui, Indrin J Chetty, Benjamin Movsas

**Affiliations:** 1 Department of Radiation Oncology, Henry Ford Health System

**Keywords:** streotactic body radiation therapy, magnetic resonance linear accelerator, retroperitoneal lesion, real-time tracking, treatment gating, hydronephrosis, planning target volume

## Abstract

Stereotactic body radiation therapy (SBRT) is an option for selected patients with metastatic disease. However, sometimes these lesions are located in such close proximity to critical normal structures that the use of safe tumoricidal SBRT doses is not achievable. Here we present a case in which real-time imaging and tracking with a magnetic resonance linear accelerator (MR-LINAC) provided a novel treatment approach and enabled safe treatment of the tumor using SBRT. Our case is a 69-year-old female who presented with localized recurrent small cell lung cancer with a retroperitoneal (FDG-avid) soft tissue lesion measuring 2.4 x 4.1 cm that was causing pain and right hydronephrosis. A Food and Drug Administration (FDA)-approved MR-LINAC system was utilized for planning and the delivery of 21 Gy in three fractions to the retroperitoneal lesion planning target volume (PTV), limited by the neighboring small bowel tolerance. The gross tumor volume (GTV) itself received 27 Gy (9 Gy per fraction). Simulation was performed using a volumetric MR imaging study in treatment position co-registered to a 4D-computed tomography (CT) image set for contouring of the target and organs at risk (OAR). Treatment planning was performed using the primary CT dataset. We developed a reasonable SBRT treatment plan to deliver the prescribed dose without exceeding tolerance doses to the right kidney, the small bowel and all other OAR’s. Real-time MR imaging and tracking during treatment delivery enabled assessment of respiratory-induced target movement in relation to the small bowel and kidney. Gating was performed to halt treatment when PTV movement exceeded the 2-mm range as specified by the treating physician. The treatment course was concluded successfully. The patient denied any acute gastrointestinal or genitourinary toxicity. The pain was significantly improved within a short time following treatment. Follow-up CT showed a near complete response of the mass with total restoration of renal functions, allowing the ureteric stent to be removed. This response has been maintained for five months till the last follow-up. In conclusion, MR-guided planning and delivery using real-time MR imaging and tracking facilitated the treatment of the retroperitoneal mass accurately and efficiently with excellent clinical and radiological response and minimal to no toxicity. We would not discern it safe to treat this mass utilizing SBRT without this ability to accurately visualize the tumor boundary using magnetic resonance imaging (MRI), and offer tracking of the target within the millimeter of surrounding critical OAR’s.

## Introduction

Small cell lung cancer (SCLC) represents approximately 14% of newly diagnosed lung cancer patients with 29,540 new cases expected in 2017 [1-3]. Although the incidence is decreasing due to decline in smoking, the female to male ratio is increasing and has reached 1:1 recently [1-3]. SCLC is a very aggressive disease with more than two-thirds of the cases presenting with extensive stage (ES-SCLC) and most of these ending with recurrences and development of widespread distant metastases [2, 4]. Even though ES-SCLC is considered incurable, two-drug platinum chemotherapy is the standard of care with robust responses and improved survival [5]. After achieving a good initial response, prophylactic cranial radiotherapy (PCI) and thoracic radiation therapy (RT) were both associated with much-improved outcomes for patients with good performance status according to the National Comprehensive Cancer Network (NCCN) guidelines [6]. Nevertheless, relapse is inevitable for the majority and second line systemic therapy in addition to palliative RT for symptomatic sites is the subsequent line of therapy [6].

Novel immunotherapeutic agents have been incorporated in the options for relapsed SCLC after the results of the CheckMate 032 clinical trial that showed improved response rates and one-year overall survival of 30 and 42% for nivolumab with or without ipilimumab, respectively [7]. However, bearing in mind the low response rate and survival outcomes associated with the second line systemic therapy for SCLC, RT can be incorporated in the palliation of metastatic symptomatic focal sites, especially for oligometastatic disease with rapid effect and subsequent enhancement in quality of life. Indirect evidence for the added benefit of consolidative RT for oligometastatic disease following systemic therapy comes from non-small cell lung cancer (NSCLC). A recent randomized study showed more than double progression-free survival benefit with the addition of stereotactic body radiation therapy (SBRT) for up to five metastatic sites in stage IV NSCLC that resulted in stopping the trial early [8].

SBRT provides a very useful tool to deliver higher doses in a short duration of time for this group of patients with limited survival and possible comorbidities [9]. Delivering high-dose fractions to targets requires robust immobilization, accurate imaging and high levels of quality assurance to ensure accurate targeting while minimizing dose to surrounding normal organs. This is more essential if the tumor is close to critical organs (within mm), which is further confounded by respiratory-induced target motion. Several fixations, imaging and tracking tools have been employed to improve target localization. On-table imaging including X-ray, cone beam computed tomography (CT) and ultrasound are used for real-time tracking. Nevertheless, all of these modalities have a quite limited capability to give an accurate actual image for soft tissue contrast compared to magnetic resonance imaging (MRI). While fiducial markers can be helpful, implantation of markers involves an invasive procedure and only provides limited information on volumetric movement of the target in real-time. Furthermore, with implanted markers, no information is provided about the location of the neighboring normal tissues nor the manner in which the target moves in relation to the normal organs in real-time. In this report, we present a case report of a patient with relapsed ES-SCLC with oligometastatic disease who achieved an excellent response to SBRT for a retroperitoneal lesion delivered using MRI-guided radiation therapy system with real-time MR-guided tracking and treatment gating.

## Case presentation

A 69-year-old life-long non-smoker female with a non-significant past medical history presented with progressive hemoptysis and a painless lump in the neck. Core needle biopsy of this lump revealed the diagnosis of SCLC and a positron emission tomography (PET)-CT and brain MRI were performed for staging. Staging workup demonstrated two FDG-avid right neck masses (2.2 x 2.1 and 1.1 x 1 cm), a 3.8 x 3.3 cm right paratracheal lesion associated with enlarged bilateral hilar lymph nodes (LN) as well as pre-carinal and subcarinal enlarged LN. MRI brain showed two metastatic foci in both cerebellar hemispheres >1 cm and the bone scan revealed a questionable metastatic focus. Accordingly, the case was deemed metastatic ES-SCLC and received whole brain RT and six cycles of first-line chemotherapy: cisplatin (80 mg/m^2^; D1) and etoposide (80 mg/m^2^; D1-3) which concluded 150 days after diagnosis with tolerable acute toxicity. Follow-up PET-CT and brain MRI showed CT partial response for the right paratracheal lesions that were totally PET-lucent with complete resolution of all other lesions including the brain metastases.

Follow-up PET-CT performed 110 days after treatment completion revealed multiple foci of increased activity in the right paratracheal region (maximum standardized uptake value (max SUV) = 6.1), a left hilar mass (max SUV = 5.2) and an enlarged right cervical LN (max SUV = 4.1) consistent with recurrent metastatic disease. At that point, RT was prescribed to areas of increased PET-CT activity and the patient received 50 Gy over 20 fractions for lung and neck lesions using intensity-modulated RT (IMRT) with Radiation Therapy Oncology Group (RTOG) grade 1 mucositis and lung toxicity. A near complete response was maintained for 4.5 months after finishing RT as per follow-up imaging.

**Figure 1:**
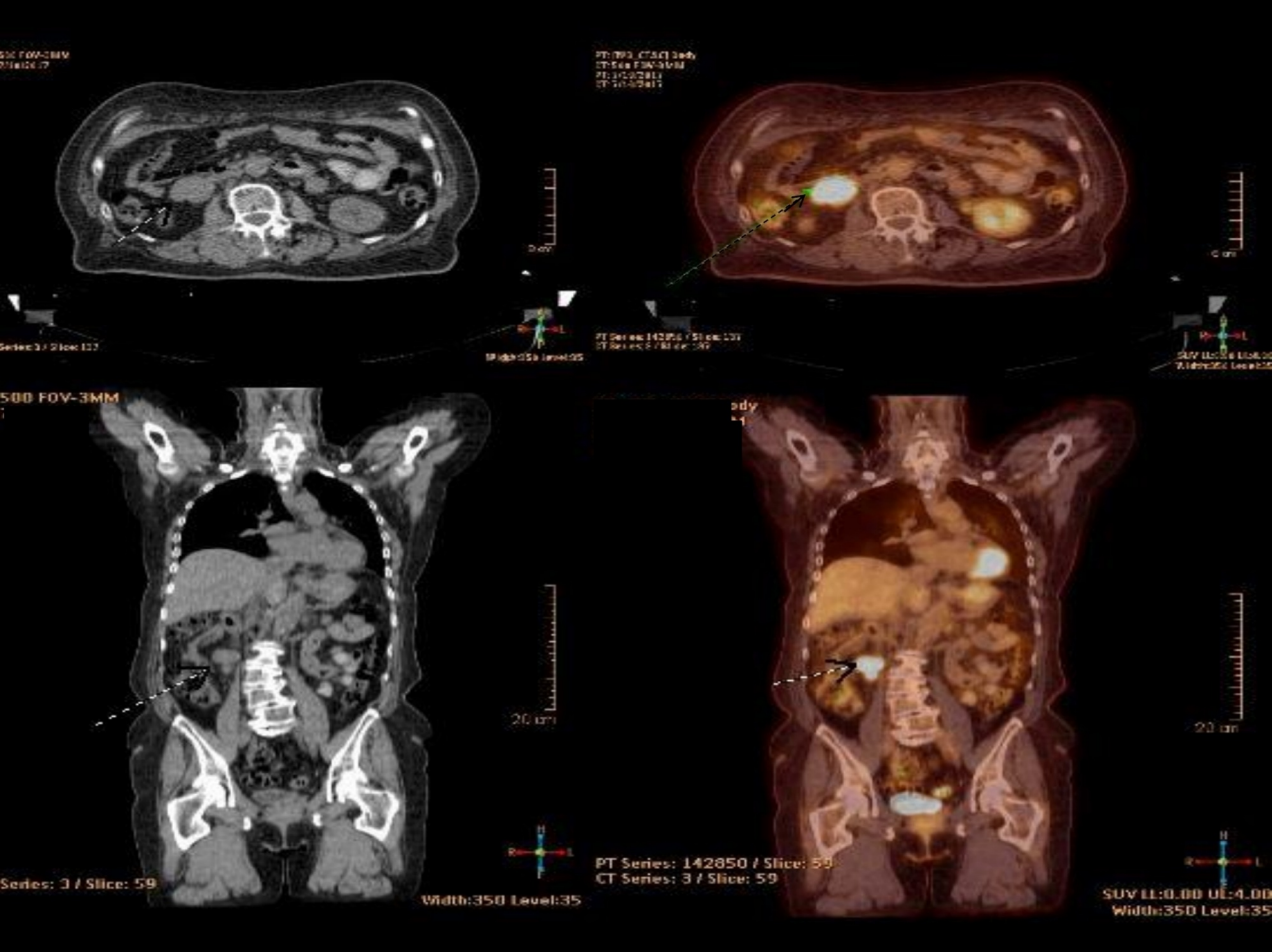
Baseline PET-CT showing the right retroperitoneal mass. PET-CT: Positron emission tomography-computed tomography

Four months after completion of RT, PET-CT demonstrated resolution of all irradiated regions with the emergence of new metastatic lesions in the left supraclavicular region (max SUV = 5.4) and right femur (max SUV = 10.6). In addition, the PET-CT showed a new 2.8 X 4.1 cm right retroperitoneal mass with highly intense FDG uptake (max SUV = 10.2) as shown in Figure 1.

This mass was painful and was associated with right hydroureteronephrosis, which contributed to deteriorated renal functions with creatinine (1.29 mg/dl), nearly double the patient’s baseline (0.71 mg/dl). This condition mandated ureteric stent placement to relieve the obstruction. Brain MRI was negative for disease progression. The decision of the multidisciplinary tumor board was taken to proceed with palliative RT for the painful femoral lesion, and subsequent SBRT to the retroperitoneal focal lesion to be followed by systemic therapy as the patient at that time was still having a performance status of 1 based on Eastern Cooperative Oncology Group (ECOG) scale. The femoral bone lesion received 30 Gy in 10 fractions.

Options for the management of the retroperitoneal mass lesion were complicated by its close proximity to the right kidney that was already having hydroureteronephrosis, as well as to the small bowel. Moreover, the respiratory-induced motion of this mass was significant. Given the patient’s co-morbidities, soft tissue lesion, and proximity of the lesion to several critical organs, we surmised that the MRI-linear accelerator (MR-LINAC) system, incorporating MRI-based imaging, and real-time tumor tracking offered the assurance of being able to deliver hypofractionated doses to this patient safely. The MR LINAC system used was the first MR-LINAC system to receive 510(k) clearance, K162393, from the US Food and Drug Administration (FDA) and has been in clinical use at our institution since July 2017. The multi-leaf collimator (MLC) constitutes a novel double-focused and double-stacked design, with banks offset by half-a-leaf width, to improve spatial resolution considerably. The 0.35 T on-board MRI scanner enables fast real-time monitoring (4 frames/second) of the tumor target, and the 6 MV, flattening filter free LINAC is equipped with a standard dose rate of 650 MU/min for efficient delivery of hypofractionated doses.

**Figure 2:**
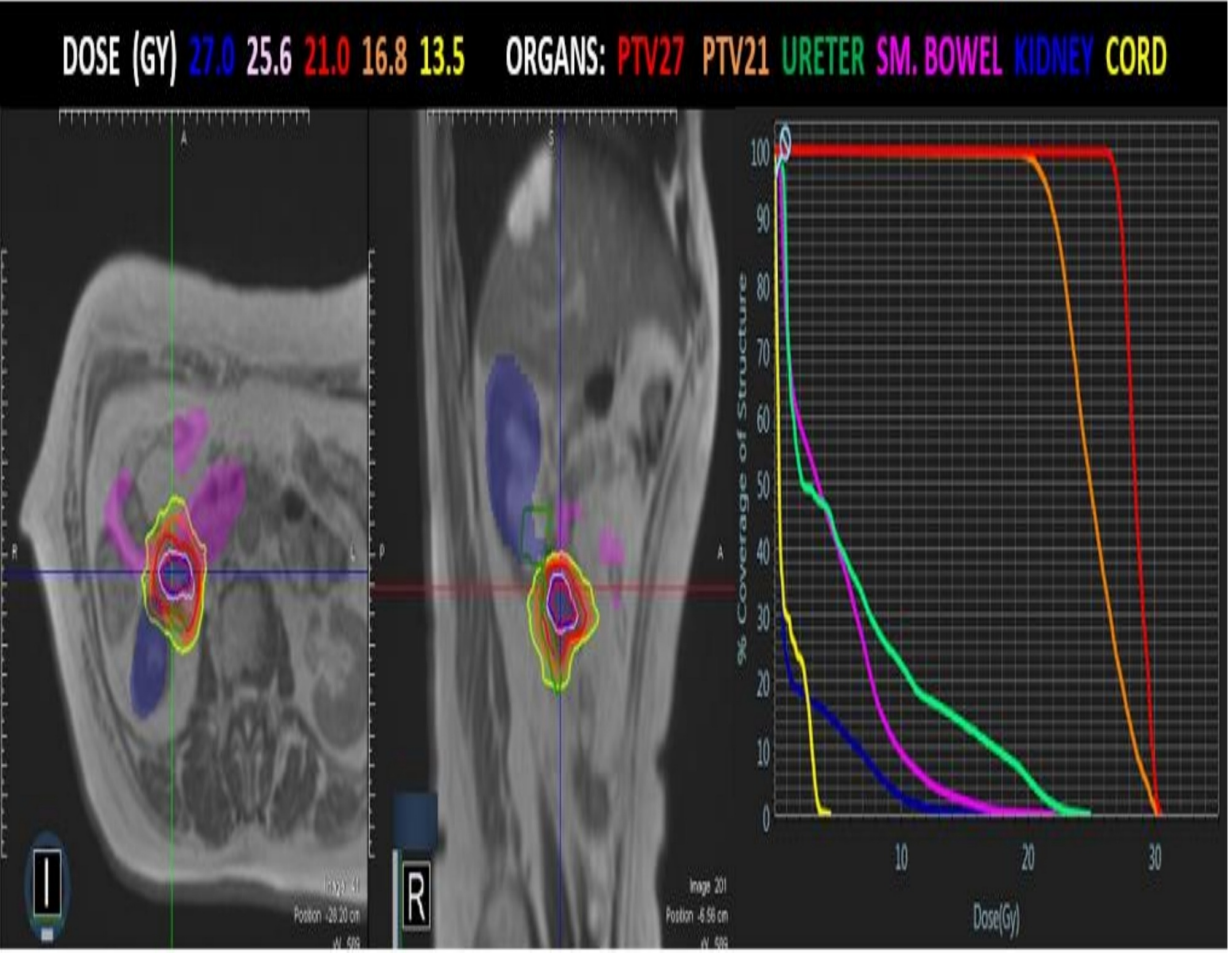
Radiotherapy treatment plan. Axial and sagittal view for PTV, OAR with isodose lines. DVH on right. PTV: Planning target volume; OAR: Organs at risk; DVH: Dose-volume histogram.

During CT-simulation, a 10-phase 4D-CT and end-exhale CT scans were acquired. The patient then underwent MR-simulation in the immobilized treatment position on the MR-LINAC. Due to lesion excursion (~1 cm in the superior/inferior direction) and proximity to the kidney and small bowel, it was determined that end-exhale breath-hold phase would be used for treatment planning, as this yielded the greatest separation between the lesion and the small bowel. Contours for gross tumor volume (GTV), planning target volume (PTV) and organs at risk were delineated and a nine-field step and shoot IMRT plan were used to deliver 27 Gy in three fractions to a simultaneous integrated boost volume receiving 21 Gy as shown Figure 2.

To deliver ~2500 MU, the total beam-on time was 4.2 minutes per fraction (~2500 MU’s), and the overall delivery time of 17 minutes. A 2-mm isotropic tracking boundary around the target was used with a 5% region of interest overlap allowed during tracking. A summary of one treatment session depicting this process is shown in Video 1.

**Video 1:** Real-time target tracking and treatment gating. Please note treatment holds (yellow square in lower left corner instead of green square) following movement of target (orange frame) outside the 2 mm margin (yellow frame) usually with respiration. Long treatment holds at 0:19-0:29 min, 0:52-1:09 min and 1:34-1:45 min.

Audio coaching was implemented to guide the patient’s breath holding.

**Figure 3:**
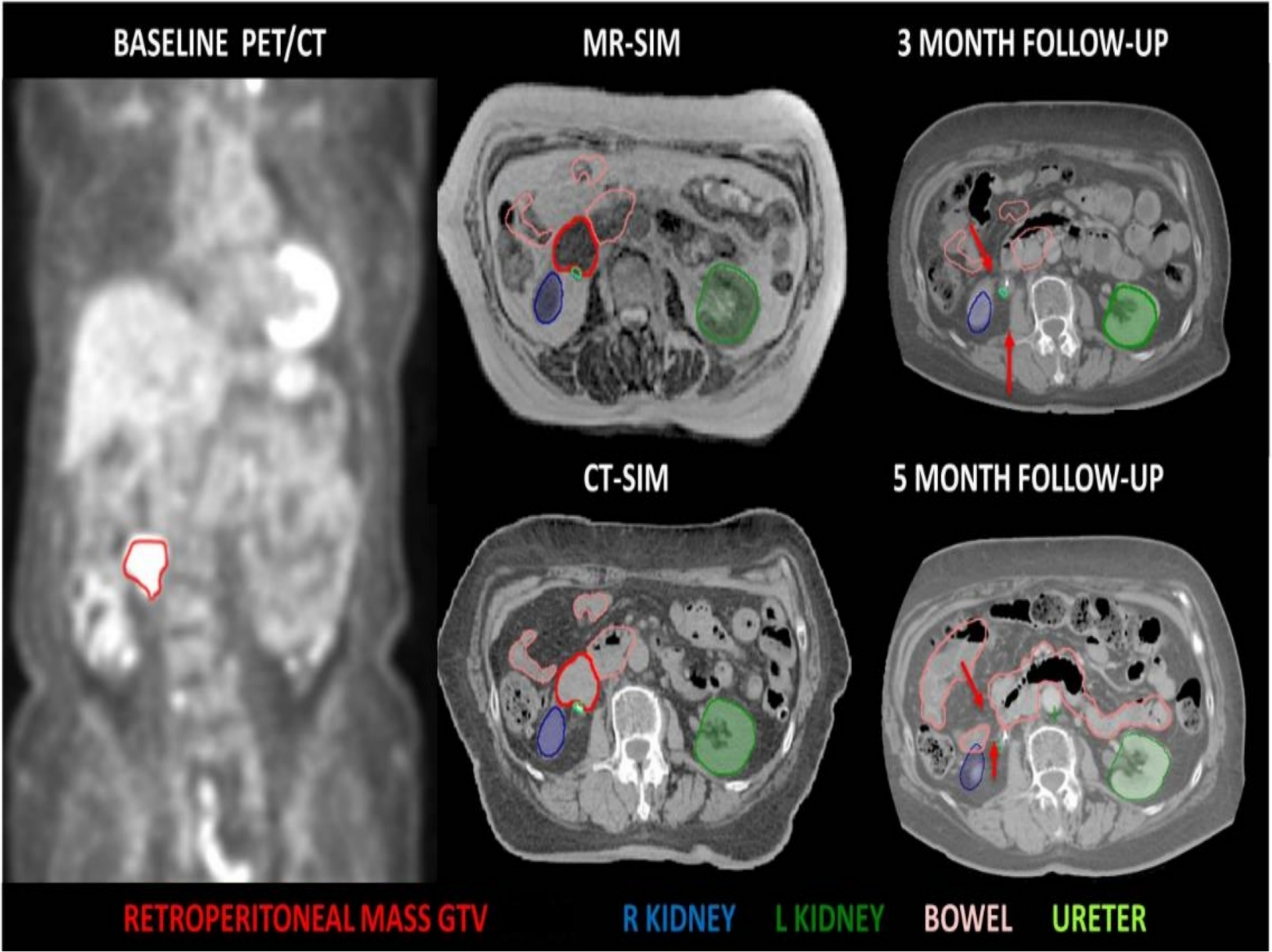
Assessment of treatment response. A comparison computed tomography (CT) scans at time of treatment simulation (middle images) showing tumor (red) in relation to kidney and bowel with CT scans three and five months after treatment (right). Red arrows mark the place of tumor showing a complete response that was maintained along five months.

After completion of RT, the patient received nivolumab combined with ipilimumab until the last follow-up. The patient denied any post-SBRT acute gastrointestinal (GI) or genitourinary (GU) toxicity in follow-up. Follow-up CT performed two and four months after the receipt of MR-guided SBRT demonstrated an interval complete resolution for the left retroperitoneal mass as well as the other lesions as shown in Figure 3.

Renal functions returned to normal, the patient had her ureteric stent removed and she is still in a good performance status (ECOG-1) with minimal use of analgesics as per her last clinical encounter.

## Discussion

Despite poor prognosis that is usually associated with ES-SCLC, this case report showed promising outcomes with the integrated use of RT, chemotherapy and immunotherapy for this patient with ES-SCLC. SBRT to the retroperitoneal mass administered after the second relapse was concluded in a markedly short time using only three fractions of MR-guided RT compared to classical fractionation using conventional techniques. This treatment achieved a rapid near complete response and allowed the initiation of nivolumab and ipilimumab within less than one month taking in consideration the lack of evidence for concomitant administration of RT with immunotherapy [7]. This resulted in a total restoration of normal renal functions that contributed to the removal of the ureteric stent and helped in maintaining good quality of life.

This case highlights the importance of real-time tracking and imaging of the soft tissue during the delivery of RT beam by MRI on table. The MR-guided technology here represents an important advancement for the treatment of these lesions close to critical organs and those that move with respiration [10]. Patient coaching and the use of abdominal compression to restrain respiratory motion are not practically suited for patients with advanced lung cancer, who often have poor respiratory functions and other comorbidities. Incorporating highly quality soft-tissue imaging using MRI, and the ability to track the target in real-time offers a much more convenient approach for these patients and enables accurate and safe delivery of relatively high doses of SBRT without increasing toxicity risk. Without this novel technology, this retroperitoneal lesion could have received a lower RT dose and inadequate coverage which would have resulted in a rather suboptimal response and potentially reduced the quality of life.

At the same time, more validation of this novel approach is warranted to improve patient localization time and treatment time. Other potential challenges include those related to planning using both MRI and CT datasets with possible disparities and relatively longer planning time. Good patient selection and accurate simulation and preparation of the patient will help in utilizing MR-guided treatment for those who are expected to attain optimal benefit of this approach. Over time, development of a learning curve and the results of randomized trials will formulate guidelines for utilizing the MR-guided RT with real-time tracking and gating.

## Conclusions

MR-guided radiation therapy with real-time tracking and gating appeared to help our patient to achieve a rapid near complete response of her retroperitoneal mass lesion with minimal to no toxicity. This great response allowed her to initiate immunotherapy in a relatively short time after the second relapse and remain in a remission with excellent quality of life until the last follow-up.
